# Persistent trophoblastic atypia on endocervical curettage found to be an epithelioid trophoblastic Tumor: A case report and review of the literature

**DOI:** 10.1016/j.gore.2022.100994

**Published:** 2022-05-05

**Authors:** Allison L. Brodsky, Marianne Hom-Tedla, Oluwole Fadare, Michael T. McHale

**Affiliations:** a University of California San Diego, Department of Gynecologic Oncology, San Diego, CA, USA; b University of California San Diego, Department of Pathology, San Diego, CA, USA

**Keywords:** Gestational trophoblastic neoplasia, Epithelial trophoblastic tumor

## Abstract

We present an unusual case of a 28-year-old female who had atypical trophoblastic proliferation on her endocervical curettage (ECC) performed at the time of a colposcopy. The indication for colposcopy was a Pap smear notable for atypical squamous cells of unknown significance, positive HPV. Initially conservative management was pursued, but given persistent atypia the patient ultimately decided to proceed with definitive management via hysterectomy. Final histologic assessment demonstrated an epithelioid trophoblastic tumor (ETT). This case highlights the unusual scenario of ETT presenting as atypical trophoblastic cells on endocervical curettage and the possible evolution of an atypical placental site nodule into an ETT.

## Highlights

• Persistent atypical trophoblastic disease found on endocervical curettage for CIN3.
• Atypia not demonstrated on repeat samplings and no abnormalities on imaging.
• Atypia recurred and final pathology demonstrated epithelial trophoblastic tumor.

## 1 Clinical presentation

This is a 28-year-old female with a history of a loop electrosurgical excision procedure (LEEP) for cervical intraepithelial neoplasia (CIN) 3 and adenocarcinoma in situ two years prior to her pregnancy. Her Pap smear during this pregnancy demonstrated atypical squamous cells of unknown significance (ASC-US) with positive co-testing for human papillomavirus (HPV). Her postpartum colposcopically-directed cervical biopsies demonstrated necrosis and hyalinization while the endocervical curettage (ECC) demonstrated atypical trophoblastic cells of the chorionic intermediate trophoblastic type. As part of her assessment due to these findings, a transvaginal ultrasound was performed. Findings were relatively unremarkable with no evidence of retained products of conception. Following the negative pelvic ultrasound, she was taken to the operating room for a hysteroscopy with dilation and curettage (D&C). Both the endometrial and endocervical curettings demonstrated a morphologically and immunophenotypically similar atypical trophoblastic proliferation. At this time, she had a negative beta-human chorionic gonadotropin (b-hCG) and human placental lactogen (hPL). A pelvic MRI was performed with no significant findings suggestive of gestational trophoblastic disease (GTD). Following this extensive and negative evaluation, she opted for close surveillance because she desired to preserve future fertility.

Approximately 4 months later, a repeat D&C was performed. Final histology did not identify trophoblastic disease. A decision was made at this time to continue to follow closely. As part of the surveillance strategy, a Pap smear and ECC were completed 6 months later. Both were negative for any significant histologic changes and as such she was counseled to continue close surveillance with her primary gynecologist. Approximately 6 months later, an ECC was repeated which again demonstrated atypical trophoblastic disease. Following this result, she opted to pursue definitive surgical management, i.e., hysterectomy. Prior to surgery, a CT scan of the chest, abdomen, and pelvis, as well as a pelvic MRI were performed. Findings from both imaging modalities were negative for any evidence of malignancy or metastatic disease. A repeat b-hCG was less than 1 mIU/mL.

The patient underwent an uncomplicated robot-assisted total laparoscopic hysterectomy and bilateral salpingectomy. Final pathology demonstrated focal epithelioid trophoblastic tumor (ETT) within the lower uterine segment. Her post-operative course was uncomplicated and she remains without evidence of disease.

## 2 Pathologic and immunohistochemical findings

**Fig. 1:**
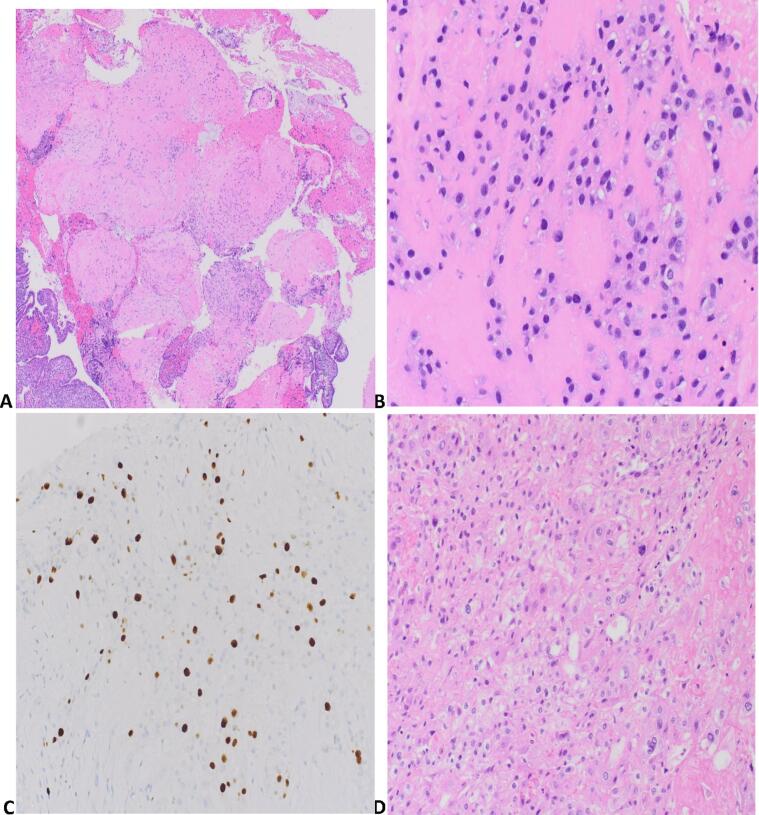
Pathologic examination of ETT. Figure 1A (Sample from curettage): Low power magnification image demonstrating aggregates of trophoblastic cells in a vague multinodular configuration. Figure 1B (Sample from curettage): High power magnification image of the atypical trophoblastic proliferation, showing epithelioid cells with eosinophilic cytoplasm and hyperchromasia in a hyalinised background Figure 1C (Sample from curettage): The lesional cells demonstrated an estimated 30% proliferation index. Figure 1D (Sample from hysterectomy specimen): Epithelioid trophoblastic tumor forming sheets of tumor cells with moderate to severe pleomorphism.

Pathologic examination of the tumor is represented in Fig. 1. The histologic findings from the multiple endocervical curettage specimens displayed similar pathologic features: multifocal, vaguely nodular to sheet-like aggregates of mildly atypical trophoblastic cells with immunoreactivity for p63 and GATA3, consistent with chorionic type intermediate trophoblasts (Fig. 1A-1C). The cells were hyperchromatic but not significantly pleomorphic. Mitotic figures were not discernible. There was an abundance of background hyalinization, necrosis and an eosinophilic fibrinous material. Ki67 showed the trophoblasts to display a proliferation index of about 30–40%. Although atypical, the alterations in these samples were not felt to be sufficiently well-developed to warrant an unequivocal diagnosis of epithelioid trophoblastic tumor.

The hysterectomy specimen demonstrated a focal proliferation in the lower uterine segment measuring 2 mm in greatest dimension with focal invasion of the uterine wall (Fig. 1D). No lymphovascular invasion was identified. The proliferation was morphologically and immunophenotypically similar to the proliferation in the prior samplings, but nuclear pleomorphism was more clearly discernible and the overall cellularity was higher. Additionally, the tumor cells were configured in sheets. This totality of findings, including the mural invasiveness, supported the diagnosis of epithelioid trophoblastic tumor.

## 3 Discussion

Gestational trophoblastic neoplasms (GTN) include a presumed derivative of chorionic-type intermediate trophoblasts (ETT), a presumed derivative of implantation-type intermediate trophoblasts (placental site trophoblastic tumor), gestational choriocarcinoma, and mixed trophoblastic tumor (WHO Classifcation, 2020). Epithelioid trophoblastic tumor is one of the rarest forms of GTN, accounting for approximately 1–2% of all GTN (Yang et al., 2019). This uncommon form of GTN was first described by Shih and Kurman in 1998 (Shih and Kurman, 1998). Two-thirds of ETT cases are preceded by a term gestation, with the other one-third of cases preceded by spontaneous miscarriage, abortion, or molar pregnancy (Gadducci et al., 2019). The mean interval between gestational event and diagnosis is 76 months (range of 2–300 months) (Mazur and Kurman, 2005). Fifty-seven to sixty-seven percent of women with ETT present with vaginal bleeding as their chief complaint. Additional symptoms include amenorrhea, abdominal pain, bloating, or symptoms related to metastatic disease. While b-hCG is elevated in 77–90% of cases, the elevation tends to be much lower than levels measured in patients with choriocarcinoma (Gadducci et al., 2019). The range of b-hCG in ETT is 12–148,460 mIU/mL with a median of 665mIU/mL (Gadducci et al., 2019, Mazur and Kurman, 2005, Zhang et al., 2013). The majority of ETTs are confined to the uterus (40%) with 31% having cervical involvement. Lastly, 25–30% have metastasis at the time of diagnosis (Mazur and Kurman, 2005).

Although FIGO is used to stage ETT, the World Health Organization Risk Score, used for choriocarcinoma, is not applicable. ETT produces less b-hCG, grows slower, has later metastases, and is less sensitive to chemotherapy compared to choriocarcinomas (Yang et al., 2019, Seckl et al., 2013). Therefore, while the treatment of ETT is often approached in a similar manner to choriocarcinoma, there are biological differences in the tumors that must be considered. The primary treatment for ETT is hysterectomy with or without pelvic lymph node sampling with few case reports of fertility-sparing treatments (Gadducci et al., 2019, Seckl et al., 2013). Some data suggests that, similarly to choriocarcinoma, an antecedent pregnancy over 4 years prior to diagnosis is a poor prognosticator, and therefore chemotherapy is also recommended for these patients (Mazur and Kurman, 2005). Patients with metastatic ETT require combination chemotherapy with subsequent cytoreduction as residual tumors can harbor microscopic disease, which is different than the treatment for metastatic choriocarcinoma (PDQ Adult Treatment, 2020). Recommended chemotherapy regimens include FAEV (5-FU, actinomycin-D, etoposide and vincristine), EMA/CO (etoposide, methotrexate, actinomycin-D, cyclophosphamide, and vincristine), and EMA/EP (etoposide, methotrexate, actinomycin-D, etoposide, and cisplatin) (Yang et al., 2019). Recent case reports have demonstrated that metastatic ETT, placental site trophoblastic tumors, and choriocarcinoma tumors with positive PD-L1 expression have responded to pembrolizumab after progression on multiple lines of chemotherapy (Bell et al., 2021, Ghorani et al., 2017, Huang et al., 2017). Speifically, a women with metastatic ETT to the lungs with a partial response to EMA/EP and > 5% PD-L1 positivity has received 29 cycles of pembrolizumab with partial response (Bell et al., 2021).

Histologically, ETT is distinguished from other forms of GTN by its distinctive composite clinicopathologic profile, as demonstrated in Table 1. Grossly, a well-circumscribed nodular mass is typical but size may be quite variable. Tumor cells may be configured in sheets, cords, or nests. Constituent cells show granular, eosinophilic to clear cytoplasm, with mostly moderate atypia. There is generally extensive necrosis, a variable mitotic index, and eosinophilic hyaline intercellular material. ETT cells are diffusely immunoreactive for HLA-G, p63, cyclin E, alpha-inhibin, and H3D3B1, with typically more limited expression of hPL and MCAM (WHO Classification, 2020, Shih and Kurman, 2004, Fadare et al., 2006, Mao et al., 2006). The Ki67-determined proliferative index for ETT is almost invariably greater than 10% (WHO Classification, 2020). ETT may colonize the cervical epithelium, thereby mimicking CIN 3 and invasive squamous cell carcinoma, especially since both entities express p63 and may be comprised of fairly monomorphic and cohesive cells with eosinophilic cytoplasm (WHO Classification, 2020, Mazur and Kurman, 2005, Shih and Kurman, 2004, Fadare et al., 2006). Our patient had a history of CIN 3 with subsequent LEEP, so squamous cell carcinoma of the cervix was in the differential diagnosis. However, other aspects of the pathologic profile excluded this possibility, including the lack of expression in our patient’s lesion of p16, a surrogate marker of high-risk HPV mediation, as would be expected in most squamous neoplasms of the cervix (Mao et al., 2006).

**Table 1:** Characteristics of subsets of gestational trophoblastic neoplasia.

	**ETT**	**SCC**	**PSTT**	**Choriocarcinoma**	**ASPN**
**Gross (macroscopic) appearance**	Circumscribed; mostly solid	Variable	Circumscribed or infiltrative; mostly solid	Variable; circumscribed or invasive; mostly soft and hemorrhagic	Mostly microscopic; less than 10 mm nodule
**Morphology**	Expansile borders; cords, nests or sheets of chorionic-type intermediate trophoblastic tumor cells with mostly moderate cytologic atypia; extensive necrosis is present; deposition of hyaline material; colonization of the epithelium may be present.	Infiltrative sheets, cords, papillae or solid nests of cells with eosinophilic cytoplasm; keratinization may be present; trophoblastic cells are absent. Mitotic activity and cytologic atypia at varying levels.	Infiltrative tumor comprised of mononuclear implantation site type intermediate trophoblasts, infiltrating between myometrial fibers and replacing vascular walls; moderate to severe cytologic atypia; admixed syncytiotrophoblasts may be present	Mononuclear cytotrophoblasts surrounded by multinucleated syncytiotrophoblasts; severe cytologic atypia; abundant necrosis and hemorrhage; mitotically active.	Nodular aggregates of chorionic-type intermediate trophoblasts, 5–10 mm in size with marked nuclear atypia. More mitotic activity, higher Ki67 index and more cellularity than is typically seen in placental site nodule
**Immunophenotype** **(diffusely positive)**	p63, GATA3, HLA-G, HSD3B1, Cyclin E, alpha-inhibin, keratins.	p16, p63, p40, keratins	hPL, MCAM, HSD3B1, HLA-G, MUC4, GATA3, keratins	SALL4, GATA3, hCG, hPL, HSD3B1, alpha-inhibin, MUC4, MCAM, keratins	p63, cytokeratins, GATA3, PLAP, alpha-inhibin
**Immunophenotype (focally positive in a subset of cases)**	MCAM, hPL, hCG,	GATA3	hCG, alpha-inhibin, p63	hPL, HLA-G, inhibin	hPL, MCAM
**Immunophenotype (Negative)**	SALL4, hCG, p16	Alpha-inhibin, PLAP, MCAM, hPL, SALL4	p16, p63, SALL4	p16	SALL4, hCG, p16,
**Ki67**	>10%	Markedly variable, generally > 50%	10–30%	>90%	5–10%

ETT- epithelioid trophoblastic tumor.

PSTT- placental site trophoblastic tumor.

ASPN – atypical placental site nodule.

SCC – squamous cell carcinoma of cervix.

In our case, histologic evaluation of the uterus confirmed the diagnosis of ETT after 25 months of surveillance for atypical trophoblastic cells initially identified on an endocervical curettage. The patient was diagnosed with an ETT two years after her antecedent pregnancy, although atypical trophoblastic cells were first noted 1 month after antecedent pregnancy. In addition, her b-hCG was repeatedly negative throughout her entire work up. The highly elevated Ki67 proliferative index for the lesion in our patient’s sampled specimens exceeded allowable levels for atypical placental site nodule (APSN), although the morphologic levels were not sufficiently developed for an overt diagnosis of ETT, especially in a clinical setting wherein a mass lesion was not apparent. In the hysterectomy specimen, which featured an invasive, albeit small lesion, an ETT diagnosis could be rendered unequivocally.

Our case is also unusual for the manner in which it was initially identified. A PubMed search of “epithelioid trophoblastic disease” yielded 61 case reports or cases series, for a total of 122 cases. There were only two cases which identified abnormal tissue on Pap smear or ECC that ultimately resulted in the diagnosis of ETT (Mao et al., 2006, Takekawa et al., 2010). In the first case, a 35-year-old female with abnormal vaginal bleeding had a Pap smear that demonstrated mononucleate, ovoid, irregular enlarged cells with hyperchromatic nuclei concerning for uterine cancer. She underwent a hysterectomy and final pathology was stage I ETT (Takekawa et al., 2010). The second case described a 42-year-old female with irregular bleeding, a Pap smear demonstrated atypical cells whose nature could not be determined (Mao et al., 2006). A cone biopsy of the cervix demonstrated an invasive epithelioid neoplasm and was diagnosed as a moderately differentiated carcinoma. She underwent a hysterectomy with bilateral salpingo-oophorectomy which demonstrated ETT in the endocervical canal and lower uterine segment. Unlike our case, both of these patients were symptomatic at time of presentation with abnormal uterine bleeding. However, asymptomatic presentations are well reported in a small subset of ETT (Pisani et al., 2021, Allison et al., 2006, Palmer et al., 2008, Davis et al., 2015).

## 4 Conclusions

We present a case that highlights the evolving clinicopathologic spectrum of ETT, a rare form of GTN. Our patient was asymptomatic, with atypical trophoblastic cells initially identified on ECC. In patients with atypical trophoblastic disease found on Pap smear, ECC, or hysteroscopy, additional diagnostic work up to assess for the possibility of GTN is warranted as is surveillance due to the indolent nature of ETT.

## 5 Informed consent statement

Informed consent to write the case report was obtained from the patient as part of standard research practices at our institution.

## Declaration of Competing Interest

The authors declare that they have no known competing financial interests or personal relationships that could have appeared to influence the work reported in this paper.
